# Understanding the Mechanism of Diabetes Mellitus in a LRBA-Deficient Patient

**DOI:** 10.3390/biology11040612

**Published:** 2022-04-18

**Authors:** Iman Hawari, Johan Ericsson, Basirudeen Syed Ahamed Kabeer, Damien Chaussabel, Asma Alsulaiti, Sanaa A. Sharari, Cristina Maccalli, Faiyaz Ahmad Khan, Khalid Hussain

**Affiliations:** 1 College of Health & Life Sciences, Hamad Bin Khalifa University, Qatar Foundation, Education City, Doha P.O. Box 34110, Qatar; pericsson@hbku.edu.qa (J.E.); sansharari@hbku.edu.qa (S.A.S.); 2 Research Branch, Sidra Medicine, Doha P.O. Box 26999, Qatar; bkabeer@sidra.org (B.S.A.K.); damien.chaussabel@jax.org (D.C.); aalsulaiti1@sidra.org (A.A.); cmaccalli@sidra.org (C.M.); faiyaz3054@hotmail.com (F.A.K.); 3 School of Medicine and Medical Science, University College Dublin, D04 V1W8 Dublin, Ireland

**Keywords:** diabetes mellitus, autoimmunity, LRBA, CTLA-4, blood transcriptomics, insulin secretion

## Abstract

**Simple Summary:**

Deficiency of lipopolysaccharide responsive beige like anchoring protein (LRBA) has been reported to cause immunological complications that can be fatal in children. Diabetes mellitus (DM) has been reported in some patients with LRBA deficiency. However, the underlying mechanism of the DM is not known. The current study provides potential novel insights into the underlying mechanism of DM in a LRBA-deficient patient. Additionally, in mouse pancreatic β-cells, we show that LRBA plays a role in the dynamics of insulin secretion and biosynthesis.

**Abstract:**

The scope of this study is to show that DM in a LRBA-deficient patient with a stop codon mutation (c.3999 G > A) was not mediated through autoimmunity. We have evaluated the ability of the proband’s T cells to be activated by assessing their CTLA-4 expression. A nonsignificant difference was seen in the CTLA-4 expression on CD3+ T cells compared to the healthy control at basal level and after stimulation with PMA/ionomycin. Blood transcriptomic analysis have shown a remarkable increase in abundance of transcripts related to CD71+ erythroid cells. There were no differences in the expression of modules related to autoimmunity diseases between the proband and pooled healthy controls. In addition, our novel findings show that siRNA knockdown of LRBA in mouse pancreatic β-cells leads reduced cellular proinsulin, insulin and consequently insulin secretion, without change in cell viability in cultured MIN6 cells.

## 1. Introduction

Diabtes mellitus (DM) is a complex heterogenous disease that is characterized by hyperglycemia due to insulin deficiency or resistance. DM imposes a heavy burden, and the incidence worldwide is increasing. DM seen in children can be classified according to the underlying cause of the disease, i.e., type 1 DM, type 2 DM, neonatal DM, maturity-onset diabetes of the young and syndromic forms of DM [1]. Precise and careful diagnosis of DM in infants is necessary in order to provide proper treatment and disease management [2].

Type 1A DM is characterized by immune dysregulation directed specifically against molecules associated with pancreatic insulin-producing β-cells (autoantigens), leading to insulin deficiency [3,4]. Proteins that were reported to be targeted by immune cells include: GAD65, insulinoma associated protein 2 (IA2), islet cell autoantigens (ICA1), pre-proinsulin, insulin B chain, islet-specific-glucose-6-phosphatase catalytic subunit-related protein and islet tyrosine phosphatase [5,6]. The autoimmune destruction of β-cells was shown to be mediated through infiltrating cytotoxic activity of the T lymphocytes in the pancreas [7,8].

Genetic alterations in Lipopolysaccharide-responsive BEACH and anchor containing protein (LRBA) are associated with early-onset common variable immunodeficiency (CVID), hypogammaglobulinemia and autoimmunity manifested in variable clinical phenotypes such as respiratory infections and inflammatory bowel disease (IBD) [9,10,11].We have shown previously that DM was seen in LRBA-deficient patients with and without detected β-related autoantibodies [12].

LRBA belongs to the family of BEACH-domain-containing proteins (BDCPs). Up to date, BDCPs consists of 9 genes, namely; lysosomal trafficking regulator (LYST), NBEA, NBEA1, NBEA2 (neurobeachin), LRBA, WDFY3, WDFY4 (WD and FYVE zinc finger domain containing), NSMAF (neutral sphingomyelinase activation-associated factor) and WDR8 (WD repeat doman 81). For the past decades, mutations in these genes have been associated with neurological, immune-related, and endocrinological human disorders [13,14]. Proteins in this family are important for membrane vesicle transport, membrane dynamics and autophagy [15].

Krummel and Allison [16] showed that CTLA-4 plays a major role in regulating the T cell immune response and maintaining immune hemostasis by suppressing the immune action. Therefore, impaired function and expression of CTLA-4 results in continuous autoreactive T cells that are manifested clinically as autoimmunity. Upon activation, CTLA-4 expression increases markedly on the surface of T cells [17]

Initial studies have provided evidence of the association of polymorphisms in CTLA-4 with T1DM [18,19]. Evidence from studies conducted on the nonobese diabetic mouse model (NOD) suggests the importance of CTLA-4 in the pathogenesis of T1DM. NOD mice are a good model for autoimmune-mediated diabetes mellitus, as the disease appears in mice aged 12–16 weeks. By that time, T cells-infiltrates (composed of CD4+ and CD8+ lymphocytes) are found on the periphery of the islets. Blockade of CTLA-4 accelerated the progression of autoimmune diabetes in NOD mice [20].

Lo [21] showed that in T cells and at the protein level, LRBA maintains CTLA-4 by preventing its’ lysosomal degradation. Patients with LRBA mutations were reported to have defects in CTLA-4 functionality and expression [22]. Recent findings show that treatment of LRBA patients with CTLA-4 mimic drugs (abatacept) was able to reverse life-threatening symptoms. Therefore, it is proposed that inflammatory and autoimmunity, including DM, in LRBA-deficient patients result from impairments and insufficiency of CTLA-4 [21].

Therefore, in this study, we aim to study the etiology of DM in a LRBA-deficient patient that was previously described [12] and to explore if LRBA has any role in insulin biosynthesis and secretion in pancreatic MIN6 β-cells.

## 2. Materials and Methods

### 2.1. Patient Recruitment

The study was approved by Institutional Review Board (IRB, reference number 1702007592) for the protection of human subjects in Sidra Medicine, Qatar. The legal guardian provided an informed written consent. Clinical details about the proband and her family were collected from the legal guardian and assigned physician in Sidra Medicine. Peripheral blood was collected for DNA, RNA and other functional assays shown in the current and previous study [12].

### 2.2. RNA Extraction and Sequencing

PAXgene Blood RNA Kit was used for the extraction and purification of total RNA from blood. After collecting the blood from the human subject in the PAXgene tube, the tube was centrifuged for 10 min at 4000× *g* to pellet the nucleic acids. The supernatant was removed, and the pellet was resuspended using 4 mL RNase-free water. The pellet was centrifuged again for 10 min at 4000× *g* and the supernatant was decanted. The pellet was dissolved in 250 μL Buffer BR1 and the sample was transferred into a 2 mL Eppendorf tube (cap left opened) and was placed into a QIAcube shaker (program: PAXgene Blood RNA PART 1). After the protocol ended, the lids were closed, and the tubes were transferred to the QIAcube shaker adapter (program: PAXgene Blood RNA Part B). After the program ended, the tubes were transferred immediately into an ice box and stored at −80 °C.

Blood transcriptome profiles were generated for 5 samples, including samples from healthy brother, the proband and 3 pooled control samples.

mRNA-sequencing was performed using QuantSeq 3′ mRNA-Seq Library Prep Kit FWD for Illumina (75 single-end) (Illumina, San Diego, CA, USA). Libraries were quantified using the KAPA HiFi Library quantification kit on a Roche LightCycler 480 (Roche, Basel, Switzerland). Cluster Generation was performed on a cBot instrument (Illumina). Samples were sequenced on Illumina HiSeq 4000 with a read-depth of 8 million.

Processing of RNA-Seq data was done using the Bcbio RNAseq pipeline (Bcbio version 1.2.3). Before alignment, a quality check of raw data was done using FastQC version 0.11.9. Alignment was done using STAR (version 2.6.1) and reads were mapped to the hg38 genome. After alignment, Samtools 1.3 was used to collect metrics on BAM files, which were further used to generate a multiQC report. FeatureCounts (version 2.0.0) was used to estimate the expression counts of the genes. Sample read counts were adjusted for library size and normalized using the Trimmed Mean of M-values (TMM) method using Bioconductor package EdgeR (version 3.34.1). Data were log2 transformed. Fold Change (FC) was calculated between patients and their respective controls using EdgeR. 

### 2.3. Data Analysis, Visualization, and Interpretation for RNA-Seq

A fixed modular blood transcriptome repertoire, BloodGen3, developed by Altman., [23] was used as a framework for data analysis and interpretation. This repertoire consists of 382 “modules or named gene sets” that were identified based on gene co-clustering patterns observed across a wide range of diseases or physiological states.

For this purpose, they used a collection of reference datasets comprised of 985 unique transcriptome profiles spanning 16 different diseases or conditions (HIV, influenza, RSV, melioidosis, *S. aureus,* tuberculosis, systemic lupus erythematosus, multiple sclerosis, chronic obstructive pulmonary disease, Kawasaki disease, juvenile dermatomyosistis, systemic-onset juvenile idiopathic arthritis, B-cell deficiency, liver transplantation, stage IV melanoma, and pregnancy). Using the weighted co-expression network and graph theory algorithm, a total of 382 modules were identified, encompassing 14,168 transcripts, from these 18 datasets.

Further, the 382 modules were organized as 38 “aggregates” (designated A1–A38), based on similarities in patterns of transcript abundance, determined this time at the module level and across the 16 reference datasets. The number of modules present in each aggregate varies between 1 and 42 modules.

### 2.4. Peripheral Blood Mononuclear Cells (PBMCs) Isolation

Peripheral blood (5 mL) from the family were collected, and PBMCs were isolated using the Ficoll density gradient separation method.

### 2.5. Antibodies

Antibodies used for human cells immunoblotting include: LRBA polyclonal antibody (Sigma, St. Louis, MI, USA, HPA023597), GAPDH (Thermofisher, Waltham, MA, USA, PA1-987), goat anti-rabbit (H + L) (Invitrogen, Carlsbad, CA, USA). For MIN6 immunoblotting, antibodies used include: LRBA polyclonal antibody (R3593-1, antibodies-online, Aachen, Germany), insulin monoclonal antibody (Santa Cruz, OR, USA, sc-377071).

Antibodies used for flow cytometry include CD8-Pacific Blue (Beckman-Coultar, Brea, CA, USA, A82791), CD4-APC Alexa Fluor 750 (A34685), CD3-VioGreen™ human (Milteni Biotec, Bergisch Gladbach, Germany, 130-113-142), CD152-FITC, human (Milteni Biotec 130-116-809), and CD279 (PD-1) human (BioLegend, San Diego, CA, USA, 329914).

### 2.6. Western Blotting

For PBMC Western blotting, 80,000 cells were used to assess the LRBA expression. The cells were lysed using M-per mammalian protein extraction reagent (Thermo Fisher Scientific Inc., Waltham, MA, USA), 1.5% Triton buffer, and Laemmli sample buffer (Bio-Rad, CA, USA). The extracted proteins were separated using Tris-Glycine gels running in SDS-PAGE buffer (Bio-Rad). Proteins were transferred to nitrocellulose membrane (Invitrogen) followed by overnight blocking with 5% fat-free milk at 4 °C. The membrane was then washed 4 times and incubated with LRBA antibody at 4 °C overnight. The membrane was then washed 3 times (5 min each) with PBS and re-incubated with horseradish peroxidase-conjugated anti-rabbit antibodies. The membrane was washed with PBS (5 min × 3) and incubated with SuperSignal WestPico chemiluminescent reagent (Thermo Fisher). The bands were detected using the ChemiDoc MP imaging system ChemiDoc Touch Gel Western Blot Imaging System; Bio-Rad, Berkeley, CA, USA) and the signals were analyzed using Image Lab software (Bio-Rad).

For cultured MIN6 cells in 6 well plates, 350 μL/sample of homemade RIPA buffer was used to lyse the cells (1% Triton X-100, 0.1% SDS and 0.5% DOC). Inhibitors added just prior to use include: PMSF (1:100) and Aprotinin (1:100). Extracted proteins were mixed with 2× Laemlli buffer and placed in a thermocycler (50 °C for one hour, or 95 °C for 5 min). The lysate was then loaded into NuPAGE 4–12% Bis-Tris gels in NuPAGE MES-SDS running buffer (Life Technologies, Carlsbad, CA, USA) and processed at 100 V for 90–180 min. Proteins were transferred to nitrocellulose membrane using wet transfer method at 380 mA for 60 min. The membranes were blocked in 5% BSA (bovine serum albumin) overnight, followed by incubation with primary antibody (diluted in 5% BSA containing 0.02% sodium azide). After washing (5 min/each) with PBST, the membrane was incubated with secondary antibody. The membrane was washed again 3 times (5 min/each) and developed using SuperSignal^TM^ West Pico Plus Chemiluminescent Substrate (Life Technologies, USA). Antibodies against housekeeping genes (GAPDH and β actin) were used as a loading control for all experiments. Bands were analyzed using Bio-Rad Image Lab software.

### 2.7. CTLA-4 Stimulation and Expression Using Flowcytometry

PBMCs were thawed at 37 °C in a water bath, and the numbers of viable cells were estimated with the Scepter 3.0 cell counter (Merck Millipore, Darmstadt, Germany) with 40 µm sensor.

1.7 × 10^6^ PBMCs were seeded in a 24-well plate in advanced RPMI media (Gibco, Thermo Fisher) supplemented with 2% human serum, 1% penicillin/streptomycin and L-Glutamine in the presence or absence of PMA-ionomycin (4 µL/mL of media). The plate was incubated in 37 °C in 5% CO_2_ incubator, overnight.

Proband’s PBMCs were used to assess the CTLA-4 expression at baseline (unstimulated), 4 and 24-h after activation with ionomycin (4 µL of ionomycin/1 ml of media). Cells were washed twice with PBS plus 2% FBS, and then surface stained with mAbs specific for CD3, CD4, CD8, CD152 (CTLA-4) and CD279 (PD-1) followed by incubation for 30 min at 4 °C. PD-1 was used as a control for immune checkpoint expression. Data acquisition was made using Navios Flow cytometry (Beckman Coulter, Miami, FL, USA) and data analysis was performed with the Kaluza software (Beckman Coulter).

### 2.8. MIN6 Cell Culture

Mouse insulinoma cell line (MIN6) was purchased from AddexBio (San Diego, CA, USA) and maintained in Dulbecco Modified Eagle Medium (DMEM, Gibco, USA, 11965-092) supplemented with 10% fetal bovine serum (FBS, Gibco, 10270-106), Sodium pyruvate (Gibco, 11360-039), antibiotic-antimycotic (Gibco, 15240-062), non-essential amino acids (MEM NEAA, Gibco, 11140-035) and 5 μL/L β-mercaptethanol in T25 flasks or 10-cm cell culture dishes.

### 2.9. RNA Extraction from MIN6 Cells and cDNA Synthesis

Total RNA was extracted from MIN6 cells sin 6-well plate using RNeasy mini Kit. (Qiagen Inc., Valencia, CA, USA). The extracted RNA was quantified using a NanoDrop Spectrophotometer (Thermo Fisher Scientific Inc., Waltham, MA, USA). 2 µg of RNA was reverse transcribed to cDNA (complementary cDNA) using SuperScript III cellsDirect cDNA Synthesis Kit (Invitrogen) and following manufacturer’s protocol.

### 2.10. Transient Knockdown of MIN6 by Small Interfering RNA (siRNA) Transfection

siRNA (20 nmole) was purchased from Invitrogen. The RNA was provided as a duplex with the following sequences, sense: 5′-CCG AGC AUU UCU UUC UGA CAU GAU U-3′ and anti-sense: 5′-AAU CAU GUC AGA AAG AAA UGC UCG G-3′. The siRNA duplex was resuspended in 1000 µL RNase-free water (molecular biology grade) to yield 20 µM and distributed in 4 aliquots and stored at −20 °C.

MIN6 cells were seeded at 70% confluency in 6-well plates. After 24 h, the media was changed to OptiMEM serum-reduced media and the cells were left in the incubator for 30 min. To transfect the cells, 50 nM of siRNA was diluted in OptiMEM media (for negative control siRNA was not added to the media). Diluted siRNA was added to diluted Lipofectamine reagent (Life Technologies) and the mixture was incubated at room temperature for 20 min to allow siRNA-lipid complex to form. The mixture was then gently added to the cells and the plate was returned to the incubator. After 24 h, the media was changed to complete media and the cells were left 48 h in the incubator.

### 2.11. Insulin Secretion Assay in MIN6

In order to assess the insulin secretion of the MIN6 cells, transfected cells were seeded one day prior to the assay in 6-well plates (≈300 × 10^3^ cells/well) with 2 mL complete medium. Next day, media was removed, and cells were washed with pre-warmed sterile PBS. Two milliliter of DMEM media (Gibco, 11966-025) was added to the cells. The media was collected, and the cells were washed with 1 mL PBS to collect residual insulin on the outer cell surface. Protease inhibitors were added to the collected media and stored immediately at −20 °C. The plate containing the cells was stored at −80 °C.

### 2.12. qRT-PCR

Gene expression (total messenger RNA levels) was quantified and analyzed using QuantStudio^TM^ Real-Time PCR system (qRT-PCR, Applied Biosystems, Waltham, MA, USA). 3 μL/sample of cDNA was added to a 96-well reaction plate (Fast, 0.1 mL, Applied Biosystems). Forward and reverse primers (primers details are listed in Table S1) were diluted to 10 µM using ultra-pure RNAase free water and mixed 1:1 and then added to the cDNA. Ten µL/sample of SYBR Green PCR master mix (Applied Biosystems) was added to the mixture and topped up to 20µL with ultrapure RNAase free water.

### 2.13. Colorimetric Assay for Cell Viability

After 24 h of transfection, cells were seeded in a final volume of 100 µL/well in a 96-well plate. Next day, 10 µL/well of tetrazolium salt WST-1 reagent was added (Sigma, 015944001). For negative control, WST-1 reagent was added to media only (without cells). The cells were incubated with the reagent for 1 h (or until media color started to change) in a humidified incubator (37 °C, 5% CO_2_). The plate was placed on a shaker for 1 min and the absorbance was measured at 420 to 480 nm, using spectrophotometer plate reader.

### 2.14. Statistical Analysis

For the flowcytometry analyses, the median between two groups was compared using Mann-Whitney U test. For the remaining assays, the normality of distribution was tested using Kolmongorov-Smirnov test. Means between groups were compared using unpaired student *t*-test. All experiments were repeated 3 times, independently.

## 3. Results

### 3.1. LRBA Is Deficient in the Proband’s PBMCs

**Figure 1:**
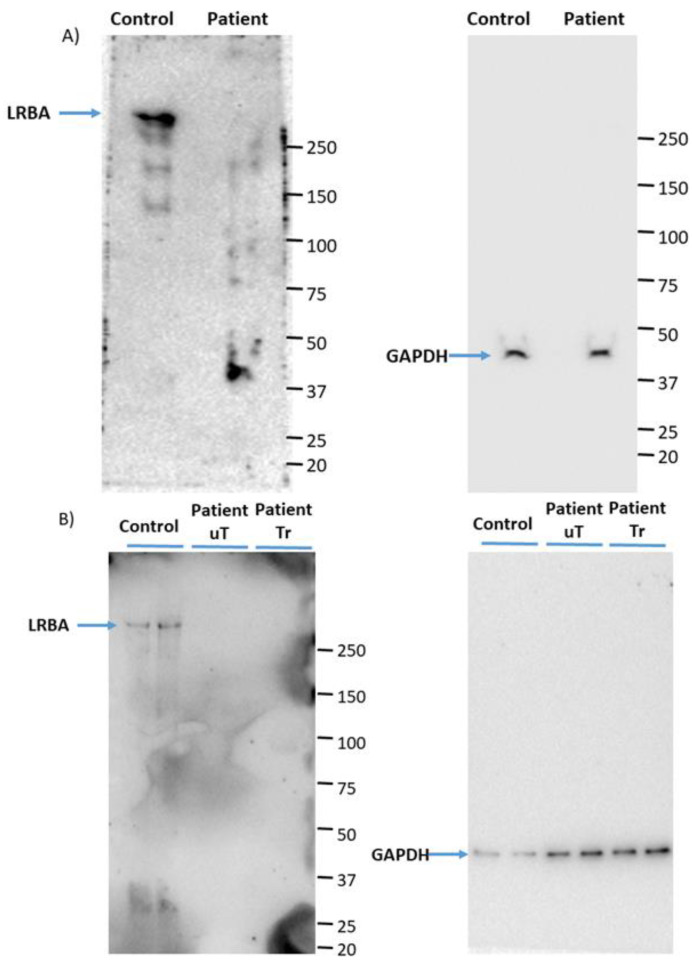
Western blots of patient PBMCs (**A**) freshly isolated from blood, and (**B**) 7 days after culturing the PBMCs in RPMI. uT (untreated) designates PBMCs isolated from blood taken before the patient was treated with Abatacept while Tr (treated) designates PBMC isolated from blood taken after the patient was treated with Abatacept. GAPDH was used as a loading control.

We have used PBMCs from the patient in order to assess the expression of LRBA. The Western blots obtained confirm that the patient is deficient for LRBA, before and after treatment with Abatacept as shown in Figure 1.

### 3.2. Normal Expression of CTLA-4 on CD3+ T Cells in LRBA-Deficient Patient

**Figure 2:**
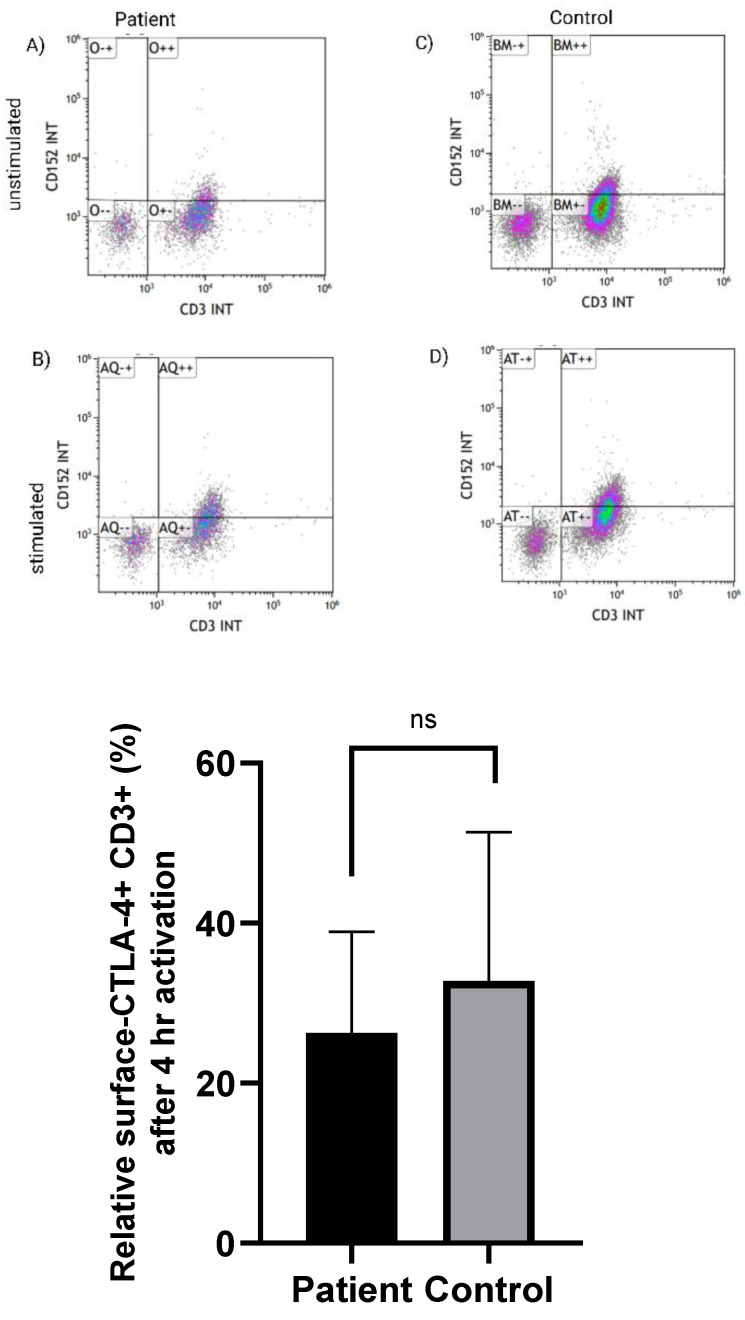
Relative CTLA-4 expression, gated on CD3+ cells. The expression of CTLA-4 on the surface of CD3+ T-cells in the proband, 4 h following activation, was none-significantly different (*p* = 0.642) compared to the control. The median between two groups was compared using Mann-Whitney U test. Error bars represent standard error of the means (±SEM). ns: non significant. Paired parametric *t* test was used. The flowcytometry images represent the CD3+ CTLA-4+ T cells in (**A**) basal level and (**B**) after 4 h of stimulation with PMA/ionomycin in the proband. (**C**,**D**) represent the flowcytometry in the healthy control at the basal and after 4 h of stimulation, respectively.

We have assessed the CTLA-4 expression on lymphocytes upon stimulation with PMA-ionomycin, a calcium ionophore, resulting in T cell activation regardless of the T-cell membrane receptor (TCR) activation signal [24]. After 4 h of stimulation, no significant differences (*p* = 0.642) were observed for the % of cells positive for CTLA-4 upon gating the cells for CD3+ T cells in the LRBA-deficient cells (Table S2 and Figure 2).

### 3.3. Immunosuppressive-Related Genes in Erythroid Cells Are Upregulated in LRBA-Deficient Proband

**Figure 3:**
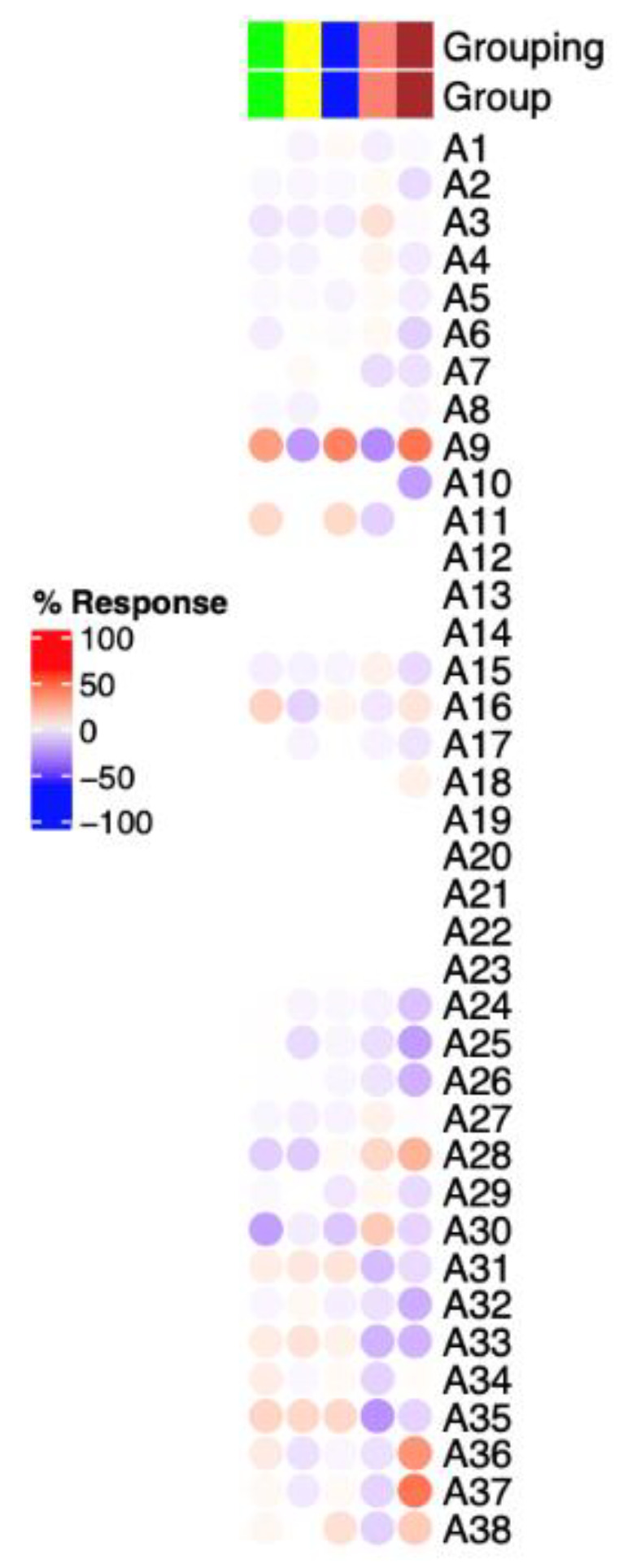
Mapping of the blood transcriptome signature of LRBA-deficient diabetic patient at the module aggregate level. The columns on this heatmap represent proband, healthy sibling and three healthy controls (from right to left). Module aggregates (A1 to A38) are arranged as rows.

An R package named BloodGen3Module was utilized to perform analyses at the module and aggregate level. Through this package it is possible to determine changes in transcript abundance at the group as well as at the individual level. The latter approach enables us to evaluate inter-individual variation within a given set of samples. The aggregate/modules with red color in Figure 3 indicates transcripts that are predominantly increased in the patient compared to pooled controls. Conversely, the aggregate/modules in green indicates those modules that are predominantly decreased.

Abundance of aggregates related to T-cells or B-cells (A1 & A2) did not differ between the proband and healthy individuals. A1 and A2 were shown to be upregulated in autoimmune diseases such as Systemic Lupus Erythematosis (SLE) and multiple sclerosis (MS).

Abundance of module A37 was highly upregulated in the LRBA-deficient patient as illustrated in Figure 3. The “Aggregate A37” consists of 11 gene modules that are related to immunosuppressive erythroid cells (list of genes in modules of interest are listed in Table S3).

### 3.4. Knocking down LRBA in MIN6 β-Cells Lead to Reduced Intracellular of Proinsulin and Insulin and Consequently Reduced Insulin Secretion

**Figure 4:**
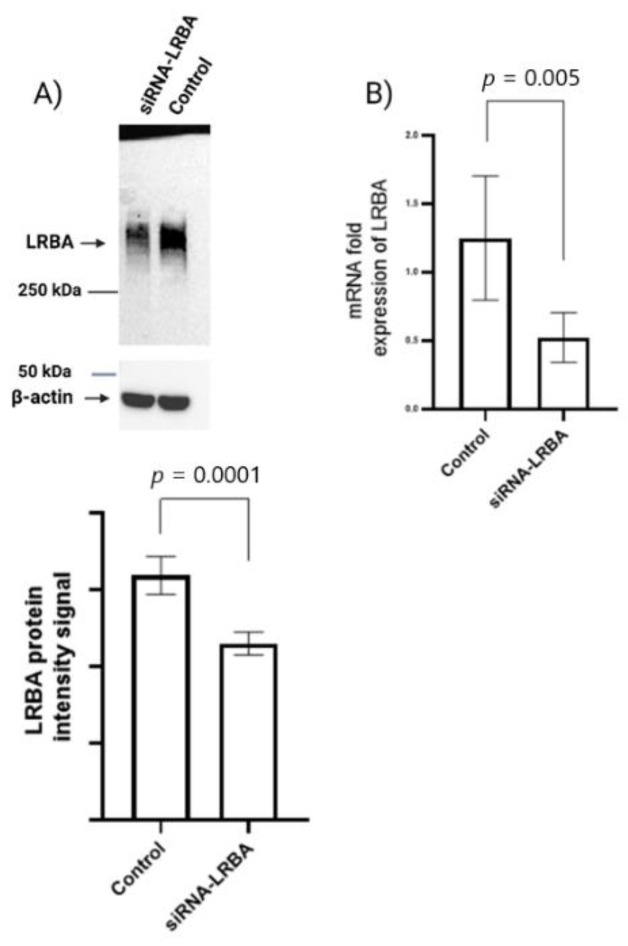
siRNA-mediated knockdown of LRBA. MIN6 cells were transfected for 72 h with siRNA targeting LRBA. (**A**) The Western blot shows successful knockdown of LRBA (at ≈319 kDa). Β-actin was used as a loading control (at ≈43 kDa). (**B**) qRT-PCR quantification of LRBA mRNA in MIN6 cells after 72 h of transfection yielded a knockdown efficiency of ≈60%, respectively. Error bars represent SEM of three independent experiments.

In this study, we are the first to report that LRBA is expressed in a mouse β-cell line, namely the MIN6 cell line (Figure 4). After 72 h of siRNA-mediated knockdown of LRBA, the cells were collected, and protein lysates prepared. As depicted, we successfully knocked down LRBA by ≈30–40% (Figure 4A). To further confirm the knockdown of LRBA in MIN6 cells, we have quantified *LRBA* mRNA levels using qRT-PCR. As illustrated in Figure 4B, the expression of LRBA mRNA was reduced by ≈60%, 72 h after siRNA transfection (Figure 4).

**Figure 5:**
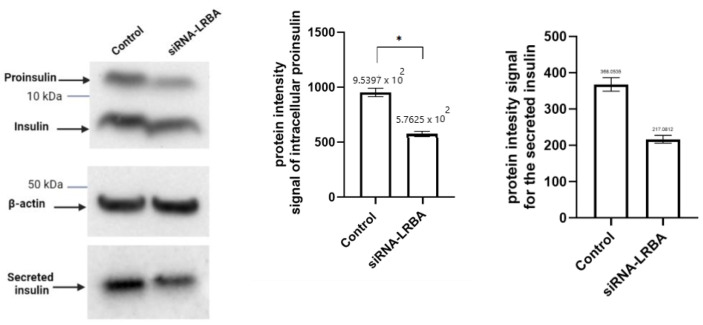
Western blots of insulin from LRBA-knockdown MIN6 pancreatic β-cells. Cells were cultured in 6-well plates and were transfected for 72 h with LRBA-targeted siRNA. Error bars represent S.D. (*: *p* < 0.05, ns: non significant). Paired parametric *t* test was used.

As depicted in Figure 5, knocking down LRBA in MIN6 cells, resulted in a significant decrease in intracellular proinsulin (*p* = 0.004), insulin and ultimately, in the secreted insulin in the media (*p* = 0.001).

### 3.5. Knocking down LRBA in MIN6 β-Cells Reduces Cellular Glucose Metabolism while Not Directly Inducing Cells Death

**Figure 6:**
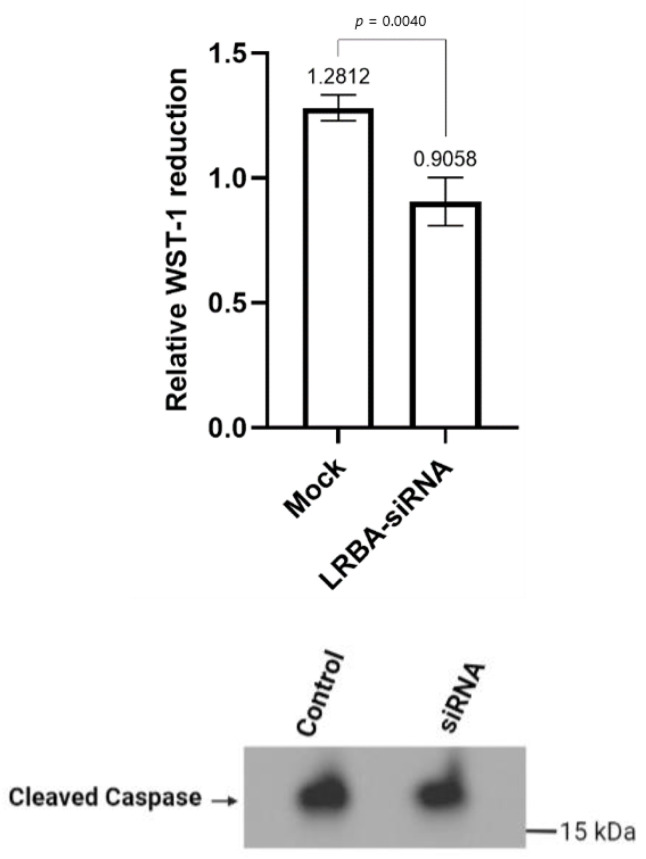
Cell viability assay in LRBA-knockdown MIN6 cells displayed a 30% reduction in cell metabolism. Cleaved-caspase-3 protein (≈17 kDa) expression was similar in control and LRBA-knockdown MIN6 cells. Error bars represent SD, the experiment was repeated three times independently with three replicates. Paired parametric *t* test was used.

The metabolic activity of the cells was monitored by WST-1 reagent in 96-well plates, 72 h after siRNA transfection. This assay assesses the cells’ ability to cleave the tetrazolium salt and form a formazan dye by the succinate-tetrazolium reductase system in the respiratory chain of the mitochondria. Knocking down LRBA in MIN6 yielded a ≈30% reduction in cellular glucose metabolism while no increase in apoptosis was observed when compared to the wildtype counterpart (Figure 6).

## 4. Discussion

Precision medicine in diabetes is defined as tailoring a treatment based on the underlying molecular mechanism of the disease. Understanding the molecular mechanism helps to characterize diabetic patients into subgroups and consequently define the appropriate treatment. Remarkably, NDM patients with mutations in *KCNJ11* or *ABCC8* being treated orally with sulfonylureas alone impacted the patients’ insulin levels in blood from undetectable to normal levels [25,26]. These early success stories of precise genetic diagnosis and the transformation of treatment from insulin injection to oral drugs have encouraged the scientific community in identifying patients with possible monogenic diabetes and understand the genotype-phenotype correlation [25].

As mentioned earlier, the child in this study was originally diagnosed with T1DM at 7 months old (due to the weakly positive GAD65 antibody). She developed symptoms of recurrent infection and hypoglobulinemia (low antibody levels in the blood). Therefore, we decided to perform whole-genome sequencing to identify the possible genetic cause of her condition. After filtering out the mutations list, a nonsense pathogenic variant in LRBA was found in exon 24 (c.3999 G > A). The wild-type codon TGG that encodes a tryptophan amino acid (W) was replaced by TGA, which encodes a premature stop codon. The pathogenic mutation obtained from whole genome sequencing was verified using Sanger method in proband’s isolated DNA. We hypothesize that the proband’s mutation leads to the degradation of mRNA through nonsense-mediated mRNA decay, as we were unable to detect any LRBA protein in patient PBMCs. Consequently, the novel mutation found in the diabetic child in this study is presumed to yield a loss of function of LRBA protein (LRBA-deficiency).

Infancy-onset DM was found in some LRBA-deficient patients with undetected β-related autoantibodies in the patients’ blood [12]. The key player, CTLA-4, which was found to be defective in LRBA-deficient patients, was hypothesized to play an important role in developing autoimmunity in these patients, such as T1DM [21]. CTLA-4 heterozygous germline mutations in human results in CTLA-4 insufficiency which is presented as complex immune dysregulation [27]. Previous studies have shown that blockade of CTLA-4 in NOD mice accelerates the onset of DM [20]. Additionally, genome-wide association studies (GWAS) have associated polymorphisms in CTLA-4 with high risk of developing T1DM [18,19].

CTLA-4 defective expression and downregulation in LRBA-deficient patients was suggested to cause autoimmunity in this group of patients [10]. To address the issue of autoimmunity in the child, we have tested the ability of the proband’s PBMCs to be activated and upregulate CTLA-4 expression on T-cells surface. CTLA-4 expression on CD3+ T-cells was none-significantly different in the diabetic proband in comparison to the healthy control. Normal expression of CTLA-4 in LRBA deficient patient was also reported before [28].

To gain a better understanding of the pathophysiology of LRBA-deficiency in the proband, we decided to investigate if there is any transcriptomic differences seen between the proband, the healthy sibling and a pooled sample from three healthy controls.

In order to facilitate the functional interpretation of RNA-seq data, we used a fixed repertoire of transcriptional modules developed by [23]. Annotations of each module and aggregates are explained in detail in the abovementioned paper. The RNA-seq data revealed that the proband had a remarkable upregulation of genes belonging to module A37 (list of genes are available in the Supplementary Materials). These genes are related to erythroid cells (CD 71+ CECs) which are immature red blood cells. Recent studies have highlighted the importance of CECs in neonates and pregnancy to suppress the immune response. Interestingly, depletion of CECs in neonate mice resulted in enhanced activation of immune cells and elevated secretion of cytokines, including IFN-Ɣ [29]. This is also manifested in human neonates where they show diminished T-cell functions, including reduced secretion of IFN-Ɣ and IL-2 upon activation [29,30,31].

Additionally, abundance of aggregates related to T cells or B cells (A1 & A2 list of genes are available in the Supplementary Materials) did not differ between the proband and healthy individuals. A1 and A2 were shown to be upregulated in autoimmune diseases such as systemix Lupus Erythematosis (SLE) and multiple sclerosis (MS).

Altogether, the data in this current study suggests that DM in LRBA-deficient patient is not an autoimmune-mediated disease.

In vitro experimental analysis of siRNA-mediated LRBA-knockdown MIN6 cell line have revealed that knocking down LRBA in β-cells yielded a decreased expression of intracellular proinsulin, insulin and ultimately decreased level of secreted insulin in the media. Knocking down LRBA in β cells have reduced the metabolic efficiency of these cells in comparison to their wildtype counterpart [32]. We hypothesize that LRBA may modulate the trafficking of insulin granules through preventing their lysosomal degradation in a similar manner seen in regulation of CTLA-4 in immune cells [21]. However, the mechanism of how LRBA affects the insulin biosynthesis and secretion needs to be studied in details in future.

## 5. Conclusions

The data presented in this study suggest that the patho-etiology of DM in some of LRBA-deficient patients is unrelated to the severe autoimmunity seen in these patients. The novelty of the current study suggests that LRBA have a role in the biosynthesis and secretion on insulin in rodent β-cell. The potential therapeutic role of LRBA needs to be deeply investigated in human samples/cell line.

The small sample size in the current study is considered a major limitation. Additionally, the current study focuses on the function of LRBA in mouse β cell, due to the infeasibility to and inability to find commercially available human β cells. Therefore it is of high importance to generate a human induced pluripotent stem cell to model DM in LRBA deficient β cell.

## Data Availability

Not applicable.

## Supplementary Materials

The following supporting information can be downloaded at: https://www.mdpi.com/article/10.3390/biology11040612/s1, Table S1: List of primers, Table S2: Flow cytometry data of CTLA-4 gated on CD3, CD8 and CD4 T cells, Table S3: List of genes of modules A1, A2 and A37.
