# Supplementary material for: Understanding the Mechanism of Diabetes Mellitus in a LRBA-Deficient Patient
Source: Biology (Basel). 2022 Apr 18;11(4):612. doi: 10.3390/biology11040612 (PMC9025338; doi:10.3390/biology11040612)
Supplement: Supplementary file 1 [file biology-11-00612-s001.zip › biology-1659992-supplementary.pdf]

Supplementary Materials

Table S1: List of primers

**Primer name**	**Primer sequence (5'→3')**	**Melting temperature (T_m)**	**Product size (base pairs)**
MS-Bactin-F	ATGAAGTGTGACGTTGACATCCGT	58.7	285 bp
MS-Bactin-R	CCTAGAAGCATTTGCGGTGCACGATG	62.1	
MSLRBA-(exon49-50)-F	GCAGGCGAGTCCATTGATGT	57.9	186 bp
MSLRBA-(exon49-50)-R	AGCAGGAAGGTTGTGCCACT	59.5	

Table S2: Flow cytometry data of CTLA-4 gated on CD3, CD8 and CD4 T cells

	PATIENT								
	CD4+ CTLA4			CD8+ CTLA-4			CD3+CTLA-4		
Baseline	0.55	3.6	2.16	2.55	9.17	5.86	3.1	12.77	8.02
after 4 hr stimulation with ionomycin	15.15	10.15	12.7	26.66	16.08	21.74	41.81	26.23	34.44
after 24 hr stimulation with ionomycin	0	4.21	2.37	0	7.1	3.65	0	11.31	6.02
	CONTROL								
	CD4+ CTLA4			CD8+ CTLA-4			CD3+ CTLA-4		
Baseline	14.66	7.61	11.135	2.3	7.78	5.04	16.96	15.39	16.175
after 4 hr stimulation with ionomycin	59.21	19.24	39.225	9.26	10.47	9.865	68.47	29.71	49.09
after 24 hr stimulation with ionomycin	16.31	8.79	12.55	8.87	4.14	6.505	25.18	12.93	19.055

Table S3: List of genes of modules A1, A2 and A37

Module A1:

EEF1B2, EEF2, LOC388524, LOC440589, LOC441246, LOC441876, LOC642250, LOC643433, LOC644029, LOC645683, LOC645899, LOC646195, LOC646200, LOC646483, LOC646766, LOC649150, LOC649447, LOC649821, LOC653232, LOC653314, LOC731096, RPL10A, RPL12, RPL17, RPL17, RPL18, RPL18A, RPL18A, RPL18A, RPL19, RPL27, RPL35, RPL35A, RPL36, RPL4, RPL5, RPL6, RPL8, RPL9, RPLP1, RPS13, RPS14, RPS14, RPS15, RPS17, RPS18, RPS20, RPS27A, RPS3, TOMM7, TOMM7

ACAT1, ACTL6A, AHCY, AIFM1, AKAP7, APOBEC3G, ARL5A, BCCIP, BCDIN3D, BMI1, BNIP3, BXDC1, BXDC2, C14ORF112, C16ORF80, C18ORF55, C1ORF19, C3ORF28, C4ORF14, C6ORF125, C6ORF130, CCDC115, CCDC50, CCT2, CNO, COPS4, CSE1L, DCTD, DCUN1D5, DDX19B, DDX21, DIMT1L, DNAJC9, EBNA1BP2, EEF1B2, EEF1E1, EI24, EIF2B1, EMG1, ENOPH1, FAM58A, FBXO21, FH, GART, GEMIN6, GLO1, GTF2H5, HACL1, HADH, HINT2, HNRNPA0, HSPE1, INTS9, JTV1, KIAA1128, KLF9, KRT10, LETMD1, MED10, METTL5, MEX3C, MIS12, MRPL15, MRPL21, MRPL32, MRPL35, MRPL43, MRPL45, MRPL46, MRPS27, MRPS30, MRPS33, NAE1, NAT5, NDUFA9, NIF3L1, NIP7, NXT1, PDCD2, PDCD5, PEPD, PFDN1, PHACTR4, PIGP, PPHLN1, PPHLN1, PRKCQ, PWP1, RAB7L1, RABGGTB, RCN2, RPAIN, RPUSD4, SCO1, SLC25A25, SNRPC, TCEAL4, TFB2M, TGIF1, TH1L, TMEM126A, TMEM203, TMEM4, TOMM20, WDR33, WDR57, WDR61, ZC3HC1, ZCCHC7, ZFP90, ZNF22, ZNF800

ABCE1, ABCE1, AGK, AKAP11, AKAP11, ANAPC1, BMS1, C19ORF2, C1ORF181, CCDC6, CDK6, CIRH1A, DIS3L, DNAJA3, DNAJC10, DPH2, ELP3, FAM62B, FBXO31, FLJ20160, FVT1, GARNL1, GFM1, GLOD4, GTPBP4, HS.213061, HS.276860, IARS, KIAA0020, LANCL1, LANCL1, LOC647346, LPAR5, LPIN1, MAP3K4, MBNL2, MKI67IP, NKRF, NOC3L, NOL11, NPAL3, NUP160, NUP205, OGFOD1, ORC5L, PPP1R16B, PRMT6, PRPS1, PTPLB, RASGRP3, RFC1, SEPHS1, SLC4A7, STAMBPL1, STK39, TP53RK, TTC19, TTC4, UBQLN4, UTP14A, WBSCR22, WDR4, WDR54, WDR67, WWP1, XPO4, ZBTB24, ZFP161, ZMYND11, ZNF146, ZNF544, ZNF816A, ZZZ3, ZZZ3

AXIN2, BCL11B, C10ORF38, CCDC104, CCNB1IP1, CD40LG, CXCR7, D4S234E, FAM102A, FAM84B, FBXO32, FCGBP, GPR18, HS.447508, HS.564504, HS.567464, KLHL3, LRRN3, LRRN3, LY9, MAGEE1, MAN1C1, MGC52498, NELL2, NMT2, NR3C2, PASK, PASK, PLEKHB1, SIDT1, SIRPG, TCEA3, TSEN54, ZNF256, ZNF548

C6ORF48, EEF1B2, EEF1G, EIF3EIP, LOC127295, LOC158345, LOC283345, LOC283412, LOC284821, LOC285053, LOC285900, LOC347292, LOC347544, LOC390354, LOC440737, LOC642989, LOC644029, LOC644511, LOC645688, LOC648622, LOC651436, LOC653232, LOC728481, LOC731985, LOC91561, RPL12, RPL13, RPL13A, RPL17, RPL3, RPS2, RPS4X, RPS4X, RPS5, RTN2, UBA52

AFF3, BANK1, BLK, CCR6, CD19, CD72, CD79A, CD79B, CD79B, COBLL1, CXCR5, E2F5, EBF1, FAM129C, FCER2, FCRL2, FCRLA, GNG7, HLA-DOA, HLA-DOB, HS.91389, LOC90925, MS4A1, OSBPL10, TCL1A, TSPAN13, TSPAN13, VPREB3

AKR1B1, ALS2CR13, APEX1, APEX1, ARL2BP, ATM, ATM, ATP6V0E2, BCL11A, BIN1, BTN3A2, C12ORF47, C12ORF57, C1QBP, C21ORF33, C5ORF39, C7ORF23, CCND2, CD2, CD47, CLNS1A, CYFIP2, DDX1, DDX18, DDX47, DENND2D, DKC1, DNMT1, DOCK10, EBI2, EEF1D, EIF3F, EIF3M, EPRS, ESD, FBXL10, FNBP4, FOXO1, FYN, GIMAP6, HLA-DQA1, ID2, ILF2, IMP3, ITK, ITM2A, LEPROTL1, LOC124512, LOC439949, LOC728554, LOC730432, LPXN, LSM5, LSM7, MCM3, MDH1, MFNG, MRPL3, MRPS24, MTSS1, MYC, NAP1L1, NCL, NSMCE4A, NUP43, OCIAD2, PABPC4, PCID2, PPIL3, PRKCH, PRKRIR, RFX5, RPAIN, RPL22, RSL1D1, SET, SHMT2, SLC38A1, SNRPA1, SNRPF, SNRPF, ST6GAL1, STARD7, STAT4, TIGA1, TINP1, TOP2B, TRAF3IP3, TTC3, UBE2N, ZMYM6, ZNF22, ZNF302, ZNHIT3, ZRANB2

AASDHPPT, ACTR6, ADO, AES, API5, ARMC1, BBS2, BCL2, BTN3A3, BUB3, BUB3, C10ORF104, C10ORF32, C18ORF17, C21ORF57, C9ORF5, CCDC25, CD69, CDC23, CDK4, CDK5RAP1, CNOT7, COPS8, CSTF3, CTSO, CUL5, CXORF26, CYP4V2, DC2, DCK, DPH5, EBAG9, EP400, EXOSC10, EXOSC6, FAM133B, FAM43A, FARS2, FLJ11171, FLJ20718, FOXK1, FUBP3, FYN, G3BP1, GEMIN4, GLS, GRPEL2, IAH1, IARS2, IBTK, KCTD6, KIAA0355, KIAA0372, KIAA0391, KIAA1826, KTN1, LAGE3, LOC203547, LOC402644, LOC644096, LRBA, LYRM2, MAGED1, MCCC1, METAP1, METTL3, MGC12966, MGC3207, MOAP1, MRPL14, MRPL50, MRPL54, MRPS27, MRPS35, MTCP1, MTERFD1, MTO1, NCBP2, NFX1, NGDN, NGDN, NHP2L1, NOP5/NOP58, NPIP, NUDT21, NUP54, NUPL2, P117, PDCD2, PPP1R2, PRKRA, PRMT1, PRMT3, PSIP1, PURA, RABGGTB, RBMX, RDH14, RNMT, RPUSD2, RYK, SDAD1, SERBP1, SFRS7, SIVA, SKIV2L2, SMAD5, SNUPN, SNURF, SNX4, SP4, SUCLA2, SUCLG2, TCEAL8, THUMPD1, TMED10, TMEM160, TMEM203, TMEM209, TMEM42, TMEM50B, UBE3A, UBE3A, UFM1, WDR75, ZFAND1, ZNF621, ZNF627, ZNF689, ZNHIT3

ADCK2, ANKRD46, ANXA6, ARL1, ATF7IP2, B3GALT6, BXDC2, C11ORF46, C19ORF12, C21ORF2, C3ORF26, C4ORF27, CCDC76, CD4, CLDND2, CSRP2BP, CSTF3, DPP7, ENTPD6, EXOSC5, FAF1, FLJ12949, FUT8, GOT1, GPR114, H2AFY2, HNRNPU, ITM2C, KLHDC4, KTELC1, LOC388564, LOC644422, LOC653479, LRPPRC, LTBP3, MALT1, MIB2, MRPL1, MRPS12, MRPS26, MUM1, NAPSA, NPIP, NUDT14, PAAF1, PAOX, PDCL3, PDCL3, PELP1, PEMT, PFAS, PHF14, PHYH, PLEKHA1, PNPLA7, POLR1C, POLRMT, PPIE, PPIE, PRMT1, QSOX2, RAI1, RPP40, RRAS2, SAAL1, SETBP1, SETMAR, SH3PXD2A, SLC25A43, SUV39H1, TBC1D4, TFB2M, TMEM116, TMEM99, TOP1MT, TRIAP1, TRMT11, TSHZ1, TSPAN3, TULP4, UFM1, WDR36, XPO4, ZBTB9, ZMYM1, ZNF17, ZNF32, ZNF337, ZNF559, ZNF827

CD247, CD247, CD3D, CD3D, CD5, CD6, CD96, CD96, DKFZP761P0423, FAIM3, GIMAP5, HS.534427, HS.554324, HS.560343, IL7R, KIAA1147, KLF12, LBH, LCK, LEF1, LRFN3, LY9, MAP4K1, PLEKHA1, PTPN4, PVRIG, SBK1, SH2D1A, SKAP1, SKAP1, SPOCK2, TNFRSF25, TRAF5, TRAT1, UBASH3A, ZAP70, ZNF831

EEF1A1, LOC387841, LOC389435, LOC401019, LOC401019, LOC401206, LOC402057, LOC440733, LOC441034, LOC642210, LOC650276, LOC652071, NACA, NACA, RPL11, RPL11, RPL3, RPL30, RPL32, RPL38, RPL38, RPL39, RPL6, RPLP1, RPLP2, RPS10, RPS11, RPS17, RPS19, RPS24, RPS25, RPS27A, RPS29, RPS29

ACO1, ALKBH3, APOBEC3F, ARV1, BBS2, BCL7C, C21ORF59, CXORF40B, DDX50, DYRK4, ELP2, EXOSC7, GSPT2, LAX1, LLGL1, LONP1, MDC1, MLLT11, MRPS9, MTERFD1, NET1, NOL8, NSUN4, PIH1D1, POLR1C, PRKCA, QDPR, RNASEH1, RNASEN, SP4, TBRG4, TCEAL3, UNG, UPF3A, UPRT, VPS45, ZNF211, ZNF644

BANF1, BOLA2, C22ORF16, C2ORF28, C8ORF59, C9ORF142, CCT8, CUTA, EIF3D, EIF3I, EIF3M, GIMAP2, HMGN1, HMGN4, HS.572219, LOC642934, LOC646849, MARCKSL1, MRFAP1L1, NDUFS3, NDUFS8, NGRN, NME1-NME2, NUP62, PIGY, POLE3, PSMG2, RING1, RPA2, SAE1, SFRS10, THAP11, TMEM14C, TUBB, XRCC6, ZNF428

AFG3L2, ALG8, ASCC3, C3ORF31, C6ORF108, COQ10A, COX10, HS.20255, KIFAP3, LDLRAP1, LOC93622, M-RIP, MAF, MEF2C, MPHOSPH1, MTSS1, NAGPA, PAICS, PARP3, PKIA, PTPLAD1, SRP72, TIMM9, TMEM134, TMEM134, TMEM5, TRIT1

ACTL6A, APOA1BP, COG2, CRYZ, CYP20A1, FLNB, FTSJ1, GPX7, HDDC3, HS.579530, LGTN, LOC642755, NDUFAF2, NOLA2, NPM3, RBM12, SCOC, SDCCAG10, SERF1B, SHPK, SIGIRR, SMARCE1, TCTEX1D2, THYN1

BTF3L4, CKS2, COPZ1, DBT, GIMAP2, GNL3, IMPDH2, KARS, LOC728492, MGMT, NACAP1, NUDT2, PCCB, PEX16, PHPT1, POLR2I, PRPSAP2, RBM34, RPL23A, SIRPG, UBE2E2, UQCRC2, UTP3, VIPR1

ABLIM1, CCR7, CD7, CDC25B, CLSTN1, FAM62A, FBL, GRAP, ITGB7, LEF1, LIME1, MAP4K1, MXD4, PTPRCAP, RFTN1, RPS23, RUNX3, SLAMF6, VEGFB

APRT, CCT7, CD79B, CUTA, GIMAP1, HNRPA1L-2, HNRPA1P4, LOC136143, LOC388275, LOC641849, LOC643997, LOC645018, LOC653820, LOC728643, RPLP0, SDF2L1, SLC41A3, SNRPN

LOC220433, LOC641848, LOC643007, LOC643949, LOC645138, LOC648343, LOC649946, LOC651202, LOC651894, LOC653658, LOC653702, LOC653773, RPL14, RPL7, RPL7A, RPL9, RPLP1

ADA, BZW2, CXXC5, DDX24, GPBAR1, IL32, LRIG1, MPHOSPH10, MRPL24, PEBP1, PECI, TBC1D9, TLR7, TMEM109, VARS, ZBTB4

ACAD11, ARL16, BAG3, C16ORF13, CD83, KIAA1128, MRPL40, PDCD4, PDS5A, PHB, POMGNT1, PPRC1, PRPF19, RNF219, SERGEF

BCAS4, FLJ35801, HOXB2, HSD17B8, LBH, LIPT1, LIPT1, LOC202134, MGC15763, MGC3020, PERLD1, SMYD3, TC2N, TMEM14A, WDR74

C6ORF48, COMMD6, DPH5, EIF3E, EIF3H, GLTSCR2, HINT1, LOC285176, LOC387867, LOC400963, LOC644039, LOC728973, RPL15, TINP1, UXT

CCDC86, ELOVL4, FBXO4, HSZFP36, INPP4B, LCMT2, MBIP, NFATC3, PDCD2L, RRP1B, RRS1, SLC25A15, TPD52, TRIM32, ZSCAN2

FAU, HLA-DRA, LOC388532, LOC388621, LOC391656, LOC731640, PFDN5, PFDN5, RPL27A, RPL31, RPS15A, RPS3A, RPS3A, RPS3A, RPS6

ABLIM1, BACH2, BEX4, BTLA, BTLA, C12ORF29, C4ORF30, C9ORF123, C9ORF45, CCDC66, CCDC91, CCNB1IP1, CCT6A, CCT6A, CD47, CD79A, CDK9, CETN3, CSTF2, DDX47, EED, FAM44B, FBXW8, GLMN, GNL3, GTPBP8, HIBADH, HS.354359, HS.436134, IER3IP1, LOC388344, LOC389641, LOC400304, LOC400986, LOC648099, MAD1L1, MRPL48, NAE1, NAPSB, PDPR, PEX6, PON2, PSMG1, RAN, RBM12, RRAS2, SLC35A3, SNORD14A, SRPK2, TAF6L, TCEA2, THOC1, TSPAN32, USP36, ZNF7, ZNF791

ATP1A1, ATP5D, BIRC3, CCDC109B, CEBPZ, CYB5B, FAM113B, GVIN1, HDDC2, HLA-DRB3, HLA-DRB6, HSPD1, IRF8, LAT, LOC387841, LOC642817, LOC643287, LOC645385, LOC648210, LOC650369, MGC3207, NAPSB, NOL5A, NUP88, PARP1, PIK3IP1, RALGDS, RWDD1, SNRPB, SNRPN, SNRPN, SSBP1, SUMF2, UBE2Q2, WDR6, WDR82

ALDH3A2, BCL11A, C1ORF57, C21ORF57, C5ORF13, C9ORF80, CSTF3, DYRK2, FAM134B, FVT1, ICOS, IDUA, KIAA1737, LOC653884, LOC728014, MCM3, MRPS17, NCAPD2, NMD3, NUP210, OSBPL7, P2RY10, PLCXD1, POP5, PRPS2, RFC4, SEC62, SETD1A, TATDN1, TCEAL8, TMEM103, TMEM106C, ZNF329, ZNF395, ZNF439

ABHD14A, C11ORF1, C16ORF30, CA5B, CD248, CD27, CD3E, CLECL1, DBP, DPH4, EBI2, FAM102A, FLT3LG, GNPDA2, GPA33, HLA-DQB1, HS.355933, HS.481464, HSF2, IL7R, ITGB3BP, ITM2A, LOC642161, LOC729101, MGC3196, PLCG1, PLCG1, RASGRP1, SCML1, SLAMF1, SLAMF6, SLC16A10, SOX8

C17ORF45, C21ORF7, DNCL1, EDF1, EIF4A2, ERP29, ETS1, FOXO3, FXYD5, HCST, HIST1H4C, HLA-DPA1, HSPA8, LOC388654, LOC440055, LOC441775, LOC652624, NDUFB8, RPL21, RPL23, RPL26, RPLP0, S100A10, S100A10, SEPT9, SPCS1, YBX1

C12ORF45, C17ORF48, C1ORF176, C3ORF17, CD320, CTPS, CXORF57, DOLK, EXOSC2, HS.374278, HS.436879, KIF5C, LOC442535, LOC728037, PARC, PTPLAD1, RNF214, RSAD1, SLC39A10, TDP1, TMEM194, TNFRSF10A, TRAM2, ZNF550, ZNF76

BUB3, C6ORF160, CECR1, EVL, GSTP1, HLA-DMA, ICAM2, ID2, LOC649049, LOC649143, MAL, MRPS6, NFS1, NOSIP, PLSCR3, RARRES3, RPA1, SNHG5, SS18L2, TMEM14B

ATG4C, ATP5A1, ATP5O, C18ORF55, C21ORF51, C3ORF10, CIAO1, CNIH, GORASP2, HPRT1, LOC145853, LOC401397, MRPL34, MRPL9, PDHB, RPS28, RPSA, TMEM14C, TRAPPC6A

ATIC, BIN1, CCND2, DCUN1D4, DDX10, EHBP1, GARNL1, HLTF, HS.145049, KTELC1, L3MBTL3, LOC492311, NUP155, PIGN, RTTN, ZBTB4, ZCCHC14, ZNF512

ANGEL1, ASCC3L1, C22ORF32, C9ORF127, DHX30, EPHX2, FBXL16, GPSM1, HS.348844, RPL37, SFI1, URG4, ZNF134, ZNF260, ZNF500, ZNF558

ADRB2, AHSA1, ATP5G2, BCL9, C10ORF32, CDK2AP2, CHCHD3, CIB1, CRSP9, ECD, EEF1A1, HSPA8, LOC400455, LOC402251, LOC440589, LOC642755, LOC645436, LOC648210, MRRF, MRRF, NME3, NOL5A, PEA15, PHF10, PIK3R1, RIOK1, RPL14, RPL24, RPL32, RPS6, SF3A2, SLC25A5, SNRPD2, TMEM126B, TMEM147, TMSB10, TUBG2, WIBG, ZC3H15

ADARB1, BCAS4, BCL11A, C12ORF24, C17ORF68, CD1C, CD52, CDR2, DCDC5, DPP7, HNRPDL, HS.546375, IL32, IRF4, LOC645968, LOC647436, LOC651453, LOC654194, LOC730525, MSTO1, NT5C3L, OPN3, PDCD7, POLR1E, PPAPDC1B, SH3KBP1, SNORD13, THOC3

ABCB1, AVPI1, BACH2, CHD6, CLCF1, CPA3, CR2, CR2, DPP4, FCER1A, FTO, HS.326560, JUP, KLRB1, KRT72, LAMA5, LPHN1, LRIG1, NDRG2, SIRT4, TSGA14, WHDC1

Module A2:

CCL4L2, CD160, CLIC3, EDG8, EOMES, FGFBP2, GNLY, GNLY, GPR56, GPR56, GZMA, GZMB, GZMH, HOPX, IL2RB, KIR2DL3, KIR2DL4, KIR3DL1, KLRC1, KLRC3, KLRD1, KLRD1, KLRF1, KLRF1, NKG7, PLEKHF1, PPP2R2B, PRF1, PTGDR, SH2D1B, TGFBR3, ZNF683

AKR7A2, ARHGAP17, ARL2, ASPSCR1, BCKDHA, BRD9, C11ORF2, C14ORF173, C17ORF61, C17ORF70, C19ORF53, C2ORF29, C6ORF153, C7ORF50, CCDC12, CCDC56, CHMP4A, CPSF4, CXXC1, E4F1, EDC4, EMD, ETFB, EXOSC1, FASTK, GPS1, GTPBP6, HARS, HCFC1, IDH3B, LOC339123, MCM7, MRPL22, MRPL37, NDUFA8, NENF, NHP2L1, NT5C, NUDC, PEX16, PLD3, PSMC5, PUF60, QRICH1, RBM14, RCC2, REPIN1, RPP21, SF3A3, SIVA, SMARCA4, SPNS1, SUSD3, TJAP1, TRAPPC2L, TRPT1, TUSC4, TXLNA, UBAC2, VIL2, YARS, ZDHHC16

ACTR1B, AK3, AOF2, AP4B1, APOL3, ASNS, ATP8B2, BEXL1, BOP1, C16ORF58, C19ORF12, C1ORF164, C3ORF37, C6ORF192, C8ORF40, C8ORF55, C9ORF114, CARD11, CCDC45, CECR5, COPS7B, CPSF1, CSNK1E, DCXR, DDHD2, DEXI, DGKA, DGKA, DMAP1, DMAP1, DNLZ, ECHS1, EIF2B5, ERCC1, EXOSC8, FAM136A, FN3KRP, GALT, GCN5L2, GPD1L, HAX1, HCST, HS.213541, IL18BP, ILF3, IMP4, ITFG2, KLHL22, LARS, LAT, LCMT1, LETMD1, LOC727820, LOC730256, LSM4, LTA, MAN1B1, MCEE, MEF2D, MRPL11, MRPL11, MRPL41, MRPS18B, MRPS21, MRPS7, MT1X, MTP18, MUTYH, NAT10, NDUFV1, NICN1, NOL1, NOL6, NSMCE1, NUDT1, PAFAH1B3, PCNT, PEX11B, PH-4, PHF15, PMPCA, PNPO, POLR2H, PPIH, PSMD8, PTGES2, PUS1, R3HCC1, RAB11FIP3, RANGAP1, RASSF7, RNF216, RNMTL1, RP9, RPL7L1, RPUSD3, SAMM50, SCAMP3, SCRIB, SF3B3, SLC25A42, SLC41A3, SMAD3, SMCR7L, SRPRB, STRA13, TH1L, TMEM138,

TMEM150, TMEM156, TOMM22, TOMM40, TSPAN31, TYSND1, UBE2G2, UCHL5IP, UQCC, WDR18, XAB1, ZC3H5, ZNF266, ZNF342

ABCF1, ABL1, ADNP, ADPRHL2, ALAD, ALKBH6, AP1B1, ASB1, ATPAF1, ATPBD1B, AYP1P1, BCL2L12, BLCAP, C12ORF62, C16ORF44, C16ORF53, C18ORF37, C1ORF123, C1ORF2, C1ORF50, C1ORF77, C20ORF27, C2ORF42, C6ORF136, CHCHD4, CLPP, CTNNBL1, CXORF40A, DPAGT1, DYNC1I2, EIF5B, ETFB, FAM116B, FLJ11286, GMDS, GTF3C6, HMG1L1, HSCB, ILVBL, KCTD21, KIAA0194, KIAA0859, LCMT1, LOC389137, LOC728564, LRRC47, MCTS1, MED29, MIF4GD, MRPL20, MRPL22, MRPL27, MRPL36, MRPS11, MRPS16, MTA2, MYL6B, NPSR1, NUPL2, PDHA1, PLEKHA9, PMM1, PRDM4, PSMC3, RABEPK, RBM4, RNF113A, RUSC1, SARS, SLC2A6, SNAPC4, SPHK2, STK25, STT3A, STX8, TATDN2, THOC4, TIMM22, TMEM189, TMEM69, TMEM93, TOR3A, TP53I13, TRAF3IP2, TRMT1, TSSC4, U2AF1L2, VARS2, ZFAND2A, ZNF362, ZNF511

ACAT2, ALG5, ALG8, ARPC5L, ATP5A1, ATP5C1, BOLA2, BOLA3, BOLA3, C14ORF166, C16ORF33, C6ORF129, C7ORF24, CCDC34, CCT7, CKS1B, COMMD1, COX5A, CUL1, CYCSL1, DDX39, DNAJB9, HNRNPAB, HNRNPAB, HNRNPD, HNRPA2B1, HSPE1, ICT1, LOC134997, LOC341457, LOC652595, LOC653505, LYAR, MIF, MRPL39, MRPS21, MRPS28, MTX2, NDUFA12, NDUFAB1, NOL5A, NOLA1, NOLA2, PGRMC2, PPA1, PRIM1, PSMA3, PSMD14, RANBP1, RANBP1, RPA3, RPL26L1, SHFM1, SSB, SYNCRIP, TCEB1, TXNL2, UCHL3, UTP11L, XTP3TPA, YWHAQ

AKR1A1, ATP1B3, ATP1B3, C16ORF61, C2ORF28, CASP3, CBLL1, CCDC32, CCDC53, CCDC59, CCDC90B, CDC16, CISD1, CLDND1, CLDND1, CNIH, COMMD3, CPSF3, CRBN, CWC15, DAP3, DDOST, DHRS4, DIABLO, EIF2B4, EIF4A3, FAM96B, FIBP, FYTTD1, GGPS1, GRSF1, HAVCR2, HSPA4, IGBP1, LOC653566, LSM3, MED6, MGST3, MITD1, MRPL18, MRPL51, MRPS22, MYCBP2, NARS, PMPCB, PPP2R5E, PRDX1, PROSC, PSMA5, PSMB2, PSMC2, PSMD7, PTPLB, SCARB2, SDHD, SELS, SF3B5, SNRPB2, STOML2, SUB1, SYVN1, TCP1, TDG, TFG, TIMM23, TMED9, TMEM85, TRAPPC4, TRIM4, TSPYL1, USO1, XPO1, YWHAG, ZBTB33

AUTS2, CD8A, CD8A, CTSW, CX3CR1, FASLG, FEZ1, FLJ20699, GFI1, GNLY, IFNG, KIAA1671, KLRG1, LAG3, MATK, MCOLN2, MLH3, MMP23B, NCALD, NCALD, PRSS23, PYHIN1, PYHIN1, PYHIN1, SAMD3, SAMD3, SAMD3, SAMD3, SYTL2, SYTL2, TARP

ABCF1, AKR7A3, ARL6IP4, BCL2L12, BRPF1, C12ORF44, C14ORF130, C16ORF24, C1ORF86, C20ORF4, CISH, COBRA1, COPS6, CRELD1, DHX30, DPM3, ERCC3, FLJ20699, GMPPA, GPR172A, GTF2F1, H1FX, HMGA1, HSD17B10, KIAA1967, LOC731314, MCM5, MGC33556, MIB2, MPV17, MRPS5, NCBP1, NDUFA10, NFATC3, ORAI1, P2RX4, PBXIP1, PHF1,

PITRM1, POLA2, PTPN7, PYGB, RCN1, RNF121, SAC3D1, SEPN1, SH3BP1, SLC25A1, SLC35A4, SLC35B2, SLC35C2, SNRPA, SSBP4, TERF2, TESK1, TMEM39B, TMEM63A, TRIM26, TSSC1, WDR46, YEATS2, ZFPM1, ZFYVE19, ZNF263, ZNF324

AGL, ATP5SL, ATPBD1B, BRD7, BRI3BP, C14ORF106, C16ORF63, C1ORF131, C2ORF30, CBFB, CRELD2, DSTN, EIF2B2, FAM116A, FRYL, GBAS, GTF3C2, HAT1, IMMT, ING3, ITGAE, ITGB1BP1, KLHL24, LOC219854, MRPS15, NCK1, PITPNC1, PLOD3, PRPF4, PTRH2, RBM17, RBM42, RNGTT, RY1, SIRT1, SMARCC2, SRP19, SS18, TMUB1, TNFSF12, UQCRH, USP42, YTHDF2, ZDHHC6

ADD3, AP1G2, BBX, C14ORF32, CKAP5, COBRA1, DCTN5, DDX56, DNAL4, EFTUD2, FAM133B, FAM53B, GOLGA3, HDAC3, HS.294603, LARP7, LMF2, MGAT2, MTDH, PILRB, PTPN11, PYCR2, RABEP1, RARS, RASSF1, RBM15, SAFB2, SDHA, SMARCD1, STARD7, SUPT16H, TCF20, TDG, TRRAP, TUFM, USP1, WDR59, XYLT2, ZFR

ACAA2, ASTE1, BLMH, BXDC5, C19ORF2, C6ORF190, CCT3, CNPY4, CRY1, GOLT1B, LOC124512, LOC644131, LTV1, MAPKAPK5, MBNL2, MRPL10, MRPL16, MRPL24, MTR, NNT, OCIAD2, P2RY10, RBBP7, SEPT11, SH3YL1, SLAIN1, TATDN1, TMEM106B, TTC27, UFSP2

C20ORF20, CANX, CANX, CLEC16A, CLTA, CNOT7, COQ5, CRLS1, DDX42, FBXW7, HSD17B4, KIAA0494, LOC440280, MED20, MGC72104, MRPS31, PBX3, PIGC, PLRG1, PPIG, RALA, RIPK5, RNF5P1, USP39, ZMAT3, ZNF277, ZNF364

CABIN1, CHM, CSNK2A2, CWF19L2, ETFDH, EXOSC10, FAM108B1, FAM98A, FLJ10769, GTF3C3, HIBCH, IQCB1, ISG20L1, KBTBD8, KLHDC5, ORC3L, POLG, PRKX, PSMA2, RASSF1, SDF4, SLC30A9, SNX5, TNRC6A, TWSG1, ZNF419

ALG3, C12ORF52, CD81, CENPB, DGCR6, DGCR6, DPP7, FASN, FKBP2, GNL1, HADH2, IL12RB1, ITPA, KRTCAP2, LOC391811, MRPS12, NSDHL, PDXP, PPM1G, TMED3, TMEM141, YIF1A, ZNF668

C10ORF88, C1ORF149, C2ORF43, ERMP1, FASTKD1, FBXL12, HSCB, LAS1L, LOC90826, MPHOSPH6, MTX2, PARP2, POLD1, PPAT, PXMP2, RPP38, RTN4IP1, SACS, SLC33A1, TSFM, ZBTB3

AGA, ARHGAP17, ATP6V0A2, ATPIF1, C20ORF100, DAZAP1, EEF2K, ENO2, LSS, LUZP1, PPP1R14A, PPP1R8, REXO4, RIOK2, RNUXA, SELS, SIRT5, SPIN4, SQLE, UCRC

C14ORF142, COMMD2, CYP20A1, DCUN1D5, DDX52, GRPEL1, HMGB1, KPNA3, LARP4, MUTED, ORC2L, PPIL5, SMARCA5, STX2, TRMT12, XRCC6BP1, ZMYM4, ZNF75

ACADM, ARID5B, ATP1A1, C20ORF55, CYBASC3, DUSP11, DUSP12, FRG1, HSPH1, KIAA0746, LOC642236, LOC653438, MRPL18, SEPW1, SRP68, TXNDC14, UBE2V2, ZNF581

ASMTL, BOLA2, C17ORF90, DNAJC8, FBXW4, FLJ21438, KIAA0409, LOC113386, MEPCE, NIT2, PARN, PRICKLE4, QARS, RNF126, RPS9, UNC84B, ZNF317, ZP3

ADRM1, AXIN1, CIAO1, CMPK1, GIMAP8, HSPD1, NCOR2, PPCS, PQLC3, RAPGEF6, SACM1L, SUMO2, SUMO3, URM1, VDAC1, WDR68

AARS, AIP, AKR1A1, C12ORF41, GNL2, IDH3B, MED30, METTL7A, NAT5, NDUFS4, SBF1, SYPL1, TMEM126B, UFC1, ZGPAT

BOLA2, C6ORF115, CCNH, CD164, GIMAP7, GPKOW, IFP38, LSM5, MAFF, NAT13, NXT2, PTMA, RNASEH2B, TXNDC9, TXNL1

ACSS1, ADAM7, ANG, ANKMY2, ANKRD55, C10ORF33, C11ORF73, C3ORF21, C9ORF105, CACNA2D3, CCL3L3, CHORDC1, CMAH, CXCR3, DHRS4, EAF2, ENPP4, FAM102B, FAM108A3, FAM119B, FLJ11795, FLJ43093, GLRX2, GRHPR, GZMM, HDHD1A, HGD, HS.31532, HS.552082, HS.568741, KIAA1147, LOC389672, LOC647037, LOC728635, LOC731950, LYSMD4, MGC57346, MS4A14, MYB, NCR3, NELF, NFKBIE, NUDT18, OSGEP, PECI, PSMD10, RDH14, SLC25A4, STT3B, TADA1L, TFPT, YEATS4, ZBTB16, ZNF142, ZNF274, ZNF593, ZNF615

ACYP2, ANKRD54, ANXA6, BIVM, BRDG1, C12ORF23, C12ORF43, C5ORF25, C6ORF66, CCL23, CHMP7, CLUAP1, CRY1, CYP2R1, DCUN1D4, DUSP14, EIF5A2, EXDL2, FAM108A3, GNGT2, HRAS, HS.513000, HS.571502, HS.66187, IFT74, IPO13, LGMN, LMTK3, LRIG2, LRRC8C, LSG1, MAGEH1, MAK10, MRPS18B, MTERF, NDFIP2, NTHL1, NUDCD2, NUP188, PARP16, PGAM5, PPAN, RDH13, RFXAP, RRP15, RUVBL1, SHFM1, SNHG3-RCC1, SUPV3L1, THUMPD2, ZNF805

AAMP, ATRIP, CCDC99, CDAN1, DAG1, DEPDC5, DET1, DUT, FAM35A, FAM50B, GATAD1, HSPC111, ICK, KIAA0090, LARP2, LDOC1L, LOC143543, LOC339804, MTAP, NARG1, NFE2L3, NFX1, NOL14, OPRS1, PET112L, PKD2, PPAPDC2, PREPL, SLC25A12, SLC35F2, TBC1D9B, TIGD5, TMEM118, TWISTNB, UPF3B, WDR12, WDR77, WRN, ZMYND19, ZNF175, ZNF480, ZNF792

ADSL, AIFM1, AMMECR1L, ASXL1, ATF5, ATP13A1, ATP5G1, ATPAF1, B4GALT7, C1ORF86, CCBL1, FANCE, FBXL6, FUK, GCDH, LOC402694, LOC647361, LSM2, MADD, MFSD3, MIZF, MRPS18A, NDUFB2, NDUFS7, PARD6A, PIGU,

PRKRIP1, PRPSAP2, PSPC1, RAPGEF1, RPS15A, RPS9, SEC61A1, SLC25A19, SLC2A8, SLC37A4, SNAPC2, TARBP2, ZBTB45, ZKSCAN2, ZNF358

ACP1, ADK, C1ORF41, CBLB, CCBL2, CCT7, CLEC2D, CSE1L, CYB5A, EDG1, EIF1AX, ERCC1, EXOSC1, FAM82C, GOT2, HNRPH1, HVCN1, ID3, KIAA0831, LOC346950, MRPL21, MRPL55, MRPL55, NUCB2, PCMTD2, PCNA, PHB2, PPP3CC, REV1, RRM1, SESN1, SYPL1, TIMM23, USP5, XRCC6, ZSCAN18

AEBP2, ANAPC10, ANKRD39, C3ORF14, CIAPIN1, CKAP2, CPSF3L, DTWD1, FTSJ2, HYLS1, KIAA1143, LOC441150, MUSK, OSBPL3, PCCA, PIGH, POFUT2, POLR2L, PRIM1, RAB33A, RAP1GDS1, RNF26, SMARCAD1, SOX4, SUMF2, UBTF, UGDH, WDR92, ZNF2, ZNF696, ZNF821

AES, ARHGDIA, ATP5D, C19ORF24, CD74, CD74, CS, EIF3G, ELK1, ERGIC3, GHDC, GPSN2, IL10RA, INTS5, L3MBTL2, MED24, NDUFB11, NUDCD3, P2RY8, PPP2R1A, RCE1, RNPS1, SMARCB1, SRM, TRIM28, XAB2, ZNF524, ZNF688

ALDH18A1, C12ORF31, CHST12, COQ3, DHRS4L2, DUT, EFTUD1, FABP5, FKRP, GPATCH4, HIRIP3, HNRNPAB, LOC642033, LOC643668, LOC650298, NHP2L1, OPA3, PAXIP1, PDIA3P, RAD51C, SCMH1, SHMT1, TCEAL3, TIMELESS, TNFAIP8L1, TTC16, TYW3

AMMECR1, C12ORF32, C14ORF179, C17ORF81, C9ORF86, CAND1, CHKA, ESD, FBLN5, FLJ20444, HEL308, HS.572538, ISOC1, KTI12, LACTB2, LOC648470, MGA, NAPEPLD, OSBPL5, PON2, RBBP9, RNF170, SLC25A26, TOB2, ZNF318, ZNF706

ASF1B, BRP44L, C10ORF57, C1ORF54, C8ORF33, DDX28, FEN1, FOXRED1, HERC2, LOC645466, MAP3K6, NUBP1, PHKG2, PRKX, RYK, SERPINB8, ST3GAL5, TCTN1, TEX264, TTF2, TUBGCP4, ZFAND1, ZNF224

ALDH16A1, AP2A2, C22ORF29, CLCN6, CRTC2, DEAF1, DOM3Z, FBXL6, GTF3C1, HS.27048, HS.485155, HYOU1, KIAA1545, MORC2, MRPS2, RAI1, RELL2, THOC3, THOC6, TP53BP1, USP21, WDR5

ALDH3A2, ALKBH7, C19ORF48, CRY2, CTSL1, CTSL1, CYB561, DHRS3, DNAJC7, GPR68, NDUFAF1, PCGF1, POLR3H, RNF5P1, RPS6KA4, SNAP29, WDR55, ZW10

ACVR2A, BPTF, C13ORF7, C21ORF33, CACNA2D4, DLAT, ESRRAP2, GK5, HS.551137, IL16, LPAR1, PALLD, PHF14, SCRN1, TAF1B, WDR12

ATMIN, BRWD2, C12ORF65, C14ORF169, EXOSC9, FCRL3, GLT8D1, GTPBP8, HSPA4, INTS2, NIPA1, PRMT6, RBM13, SBDS, SFRS12, TAF1B

C19ORF61, CDCA7L, CTBP1, EI24, HS.558212, LOC643790, MRPS34, NOLA1, NR2C1, PINX1, SLC12A2, TARBP2, TMEM41A, TSR1, TULP4, USP14

C6ORF66, CCDC28B, CHCHD7, KIAA0495, LOC137107, PBX4, PNOC, PNRC2, RLTPR, RPL23AP13, SIRPG, SNORD68, TMEM50B, WDR21A, ZNF280C, ZNF627

ACN9, AHNAK, C1ORF212, C20ORF7, HNRPUL2, HSP90AA1, LOC648000, LOC653226, OCIAD1, OXR1, SFRS3, SLC35B3, SRP9, THYN1, TOMM70A

ACAD10, ALG9, ANKRD27, ATXN10, BCAP29, BYSL, C10ORF61, C12ORF30, C17ORF39, C17ORF59, C17ORF81, C19ORF29, C1ORF109, C3ORF26, C5ORF3, CAPN10, CCDC127, CDCA4, CHCHD8, CLUAP1, COMTD1, DCTD, DDX26B, DDX31, DFFA, DRG2, EIF1AX, ELP4, ENDOG, EXOC2, EXOC2, FAM26B, FARSA, FASTK, FBXW9, FMO4, GAMT, GATA3, GNB1L, HDHD3, HS.40289, HS.430851, HS.508889, HS.556082, IDH3A, INPP5B, IPO9, KIAA0125, KIAA0195, KIAA0460, LANCL2, LIG3, LIMA1, LOC653071, LOC729985, LOC90120, LRRC37A4, MADD, MAPKAPK5, MARS2, MBIP, METTL1, MIB1, MORG1, N6AMT2, NBPF14, NCAPD3, NMT1, NOB1, NOLC1, NUP54, NUP62, OTUD6B, PALB2, PDIA4, PFKM, PHC1, PMS2L5, PNKD, POLA1, PPIL1, PRKAB2, PSMG3, RCL1, RNASEH2A, RPL39L, RUFY3, SEH1L, SFMBT1, SHQ1, SLC25A4, SMC2, SMYD4, SNX25, SYMPK, TCEAL1, TCERG1, TCF3, TPP2, TRAF1, TSGA14, TUT1, UQCR, USP11, WDR33, WDR60, WDR79, XPO5, ZBTB25, ZBTB5, ZBTB8OS, ZC3H8, ZCCHC3, ZCWPW1, ZNF618, ZNF74, ZNF831

ALDOC, ATP2A2, C11ORF24, C16ORF53, C7ORF44, C8ORF53, CCDC101, CHD1L, CIRBP, CLCN6, CNOT2, DVL2, GNB2L1, HMG1L1, IFI6, LCMT1, LEMD2, LOC143543, LOC391356, LOC440927, LOC643284, LOC645317, LOC647000, LOC647000, LOC653314, LRRC8D, NUTF2, OTUB1, OXA1L, PEX5, POMT1, PQBP1, PQBP1, RER1, RNF126, RNF41, RPL37A, RPS16, RPS27, SEC11C, SNRPB2, TBL2, TCEAL8, TIMM8B, TMEM185A, TUBB, TYW1, UBE2Z, YAF2, ZNRD1

ABHD14B, ABI3, APRT, C17ORF87, C8ORF33, CRIP1, CRYL1, EIF2S1, ETS1, FOXP1, GGA2, GTPBP3, HLA-DMB, HLA-DRA, HS.505676, INF2, ITCH, MAGED1, MAL, MAP3K7IP1, NPAT, PAPD1, POLM, PRNPIP, PTPRO, RBM45, RTN1, SLC35C1, SMYD2, SP140, ST3GAL5, TGIF2, UCHL5

Module A37:

MARCH8, ALAS2, ALAS2, BCL2L1, BPGM, C14ORF45, C1ORF128, CA1, EPB42, FAM46C, FECH, GMPR, GPR146, GYPB, GYPE, IFIT1L, KRT1, NFIX, OR2W3, OSBP2, PLEK2, SELENBP1, SLC14A1, SLC4A1, SNCA, TMCC2, TMOD1, TNS1, TRIM10, TUBB2A, XK

ABCC4, AMFR, BAT3, BMP2K, C17ORF39, C9ORF40, DNAJB2, EIF1B, GDE1, GNA12, HS.211743, HS.57079, IQWD1, JAZF1, MED25, NSUN3, ODC1, POLR1D, PPP2R5B, PSME4, RANBP10, RNF14, RSRC1, SELK, SLC2A1, SNX3, TBC1D22B, TMEM183A, TMEM183B, UBE2F, UBQLN1, WBP2, WDR26, WDR45, WDR51B, WNK1, ZBTB44, ZER1, ZNF653

ASCC2, C18ORF10, C18ORF10, DPM2, EPB49, FBXO7, FBXO7, GATA1, HAGH, HEMGN, HEMGN, HMBS, KEL, LOC284422, LOC440359, LOC441081, MBNL3, MBNL3, MYL4, PDZK1IP1, PHOSPHO1, RUNDC3A, SESN3, TESC, TGM2, TRIM10, TSPAN5, VWCE

AP2M1, AP2M1, AP2S1, ATG9A, ATG9A, B4GALT3, BCL2L13, C13ORF15, C19ORF62, CYB5R3, DAP, DENND1A, FAM134A, HK1, JUND, MSI2, NTAN1, NUCB1, PA2G4, POLR1D, TCEB2, UBAC1, UBAP1, UBL7, UROD, WDR13, WIPI2, ZMAT2

ALDH5A1, ALDH5A1, BNIP3L, BNIP3L, C20ORF108, DNAJA4, FAM104A, HBD, HS.105618, HS.291319, ISCA1L, LOC389293, MPP1, MXI1, PRDX2, PRDX2, RAB2B, RAD23A, RIOK3, RPIA, SIAH2, SLC1A5, TFDP1, TFDP1, TRAK2, TSTA3, UBE2O, YOD1

AP2A1, ATP6V0C, BMP2K, C16ORF35, C19ORF22, C2ORF24, C9ORF78, CCDC23, CREG1, EIF2AK1, ELOF1, FAM100A, FOXO4, FURIN, GABARAPL2, GCLC, GPX1, H1F0, HDGF, ISCA1, ISCA1, LYL1, MAF1, MKRN1, NINJ2, NP, PIM1, PINK1, PNPLA2, PRR6, PSMF1, RFESD, RNF123, RNF14, STK33, STOM, SYT15, TERF2IP, TFDP2, XPO7, YPEL3

ABCC13, ADIPOR1, ARL4A, BLVRB, BOAT, C16ORF35, CARM1, CES3, CSDA, FBXO9, FIS1, FKBP8, FLJ20489, GLUL, GSPT1, GUK1, GYPC, GYPC, HAGH, HBM, HBQ1, HPS1, HPS1, PBX1, PTMS, SHARPIN, SLC25A39, ST6GALNAC4, ST6GALNAC4, TMEM86B, UBL7, UBXD1

MARCH8, BCL2L1, BSG, CDC34, CHPT1, CHPT1, FHL2, GLRX5, IGF2BP2, KLF1, LOC650832, LOC653778, LOC654103, MAP2K3, RBM38, RBM38, RIOK3, TMEM63B

BRD4, C16ORF35, CDKL1, DPM2, EPB41, HMBS, HPS1, IQWD1, KLC3, LOC643008, LOC648434, LOC650898, MAP2K3, MAP2K3, MICAL2, MICALCL, MXI1, MXI1, RAB3IL1, RP11-529I10.4, SLC38A5, SLC6A8, TCP11L2, TGM2, TMPRSS9, TTC25, WNK1

ANKRD9, ATP6V0C, C22ORF25, C5ORF4, E2F2, FHL2, HMG2L1, LGALS3, LOC653907, MAP2K3, MARCH2, MCOLN1, MGC13057, PPM1A, PPM1A, RNF10, RNF11, SLC6A10P, SMOX, SRRD, TMEM158, UBE2H

ARHGEF12, C14ORF45, CISD2, CMBL, FLCN, GCAT, GYPE, HBBP1, LOC253012, LOC253012, PCSK1N, PLVAP, RHD, SLC6A9, SPTB, TBCEL, TMEM56, YPEL4
